# Increased Oxidative Stress in Gastric Cancer Patients and Their First-Degree Relatives: A Prospective Study from Northeastern Brazil

**DOI:** 10.1155/2021/6657434

**Published:** 2021-11-27

**Authors:** Manuel B. Braga-Neto, Deiziane V. S. Costa, Dulciene M. M. Queiroz, Felipe S. Maciel, Michelle S. de Oliveira, Antônio B. Viana-Junior, Flávia A. Santos, Renata F. C. Leitao, Gerly A. C. Brito, Paulo R. L. Vasconcelos, Lucia L. B. C. Braga

**Affiliations:** ^1^Department of Internal Medicine, Federal University of Ceará, Fortaleza, CE, Brazil; ^2^Division of Gastroenterology and Hepatology, Mayo Clinic, Rochester, MN, USA; ^3^Institute of Biomedicine for Brazilian Semi-Arid and Clinical Research Unit/Federal University of Ceara, Fortaleza, CE, Brazil; ^4^Postgraduate Program in Morphofunctional Sciences, Department of Morphology, Federal University of Ceara, Fortaleza, CE, Brazil; ^5^Laboratory of Research in Bacteriology, Universidade Federal de Minas Gerais, Belo Horizonte, MG, Brazil; ^6^Department of Physiology and Pharmacology, Federal University of Ceará, Fortaleza, CE, Brazil; ^7^Department of Surgery, Federal University of Ceará, Fortaleza, CE, Brazil

## Abstract

**Background and Aims:**

First-degree relatives of gastric cancer patients are at increased risk of developing gastric cancer. Increased oxidative stress, including lipid peroxidation, has been associated with gastric carcinogenesis. Whether first-degree relatives of gastric cancer patients have increased oxidative stress remains unknown. We aimed to compare oxidative stress in patients with gastric cancer, their first-degree relatives, and dyspeptic controls.

**Methods:**

A total of 155 patients undergoing upper endoscopy were prospectively enrolled, including 50 with gastric cancer, 49 first-degree relatives of gastric cancer patients, and 56 controls. Serum concentrations of malondialdehyde (MDA) and glutathione) and activities of superoxide dismutase (SOD) and catalase were measured. Multivariate analysis adjusting for sex, age, smoking status, and alcohol consumption was performed.

**Results:**

Lipid peroxidation, as measured by concentration of MDA (nmol/mL), was higher (*p* = 0.04), and glutathione levels were lower (*p* < 0.001) in the gastric cancer group compared to controls. There was no difference in the catalase activity among the groups. There was no difference in glutathione and MDA concentration or catalase activity between the different stages of gastric cancer based on the TNM classification. Relatives of gastric cancer patients had higher glutathione concentration (*μ*mol/mL) compared to gastric cancer patients (262.5 vs. 144.6; *p* = 0.018), while there was no difference in MDA concentration. Catalase and superoxide dismutase activity were lower in the gastric cancer group (3.82 vs. 0.91; *p* < 0.001 and 1.04 vs. 0.6; *p* < 0.001) compared to their first-degree relatives. Interestingly, MDA concentration in the first-degree relative group was higher than in the control group (7.9 vs. 5.1; *p* = 0.03).

**Conclusions:**

In this study, similarly to gastric cancer patients, their first-degree relatives were found to have increased oxidative stress compared to controls. Further studies are warranted to validate this observation and to better understand the role of oxidative stress as a possible biomarker in this population.

## 1. Introduction

Gastric cancer (GC) is the fifth most common malignancy worldwide and the third most frequent cause of cancer-related mortality [1]. The overall five-year survival for gastric cancer is approximately 18% [2]. In Brazil, GC is the fourth and sixth more incident malignancy, respectively, among men and women (INCA 2020) with great variation of the incidence among regions and between states in the same regions. In Northeastern Brazil, the State of Ceará has the highest incidence of GC, and it has been estimated that 2020 annual adjusted incidence for men is 18.19 per 100.000 [3] (INCA 2020), compared to an overall incidence of 9.3 per 100,000 in Brazil. In Japan, the annual incidence is 29.6 per 100,000, while in the United States, this rate is much lower at 6 per 100,000 [4]. The pathogenesis of gastric cancer is multifactorial, including genetic and environmental risk factors. A large number of evidence indicates that reactive oxygen species (ROS) are associated with the process of carcinogenesis by damaging the structure of DNA and tumor suppressor genes [5–7].

Reactive oxygen species regulate cellular homeostasis and are produced in response to several conditions such as ultraviolet radiation, smoking, alcohol, NSAID use, and chronic inflammation, such as seen in *H. pylori* infection [8–10]. Antioxidants limit the toxicity associated with free radicals. Superoxide dismutase and catalase are antioxidant enzymes that neutralize ROS by converting superoxide (O_2_^−^) into hydrogen peroxide (H_2_O_2_) which is then converted into H_2_O and O_2_ by catalase. Glutathione reductase removes H_2_O_2_ by oxidizing reduced glutathione (GSH), a major nonenzymatic antioxidant, to oxidized glutathione [8, 11]. The imbalance in the generation of ROS and detoxification produces oxidative stress resulting in lipid peroxidation, and polyunsaturated fatty acids are converted to malondialdehyde (MDA) which can then lead to DNA damage and carcinogenesis [12]. ROS and reactive nitrogen species (RNS) along with lipid oxidation cause protein oxidation which gives rise to protein carbonyls (e.g., aldehydes and ketones). It has been suggested that oxidative stress parameters could be valuable in monitoring cancer occurrence and progression of the cancer [13, 14]. Although most studies suggest that oxidative stress is increased in gastric cancer [15, 16], others have not found such an association [17].

Several factors have been involved in the pathogenesis of GC, including *H. pylori* infection (considered to be the strongest factor), genetic susceptibility, smoking, dietary habits, and environmental factors [18]. First-degree relatives of GC patients are known to be at 2- to 3-fold higher risk of developing the disease [19], which might be due not only to genetic factors but also to infection by more virulent *H. pylori* strains [20, 21]. However, it remains unknown whether first-degree relatives of GC are more predisposed to ongoing oxidative stress than individuals without a family history of GC.

Therefore, the aim of this study was to investigate the oxidative stress by evaluating catalase and superoxide dismutase activity and levels of glutathione and MDA in the serum of patients with GC, as well as in first-degree relatives of GC and dyspeptic controls without a family history of gastric cancer.

## 2. Methods

The study was approved by the Institution's Ethics Committee of Research of the Federal University of Ceará (approval number: 628.750), and all patients signed an informed consent form. The patients were selected among those seen at the Federal University Ceara's Walter Cantideo, part of the Hospitals of the Public Health System (Sistema Único de Saúde) that provides health care to low-income subjects. The patients have similarity in respect to the ethnic background, social economic level, area of residence, and sex. Clinical symptoms and demographic data such as age, sex, place of residence, alcohol consumption, and tobacco use were obtained by a questionnaire answered by all patients. The patients were enrolled from 2014 to 2015, and all of them were interviewed face-to-face. The study sample population was selected on inclusion and exclusion criteria.

### 2.1. Selection of Patients (Inclusion and Exclusion Criteria)

The diagnosis of non-cardia GC patients was confirmed by histopathology according to the classification of Lauren [22]. Patients with gastroesophageal junction tumors, non-Hodgkin gastric lymphoma, or gastrointestinal stromal tumors were not included in the study. The GC patients were not receiving chemotherapy or radiotherapy at the time of the study. The staging of GC was evaluated by the TNM classification as suggested by the American Joint Committee on Cancer (AJCC) [23].

Asymptomatic first-degree relatives of GC patients were invited to participate in the study and underwent an upper gastrointestinal endoscopy with obtaining gastric biopsies. Controls were patients with dyspepsia without GC family history (CG) who underwent upper gastrointestinal endoscopy for investigation of their dyspepsia at the Hospital Walter Cantideo. Patients with chronic disease such as liver, pulmonary, renal, cardiac, peptic ulcer disease, hematologic, neurological, metabolic, endocrine, or autoimmune disorders were not included. Patients with history of gastric surgery, active gastrointestinal bleeding, use of steroids, and immunosuppressive drugs were not included in the study.

### 2.2. Processing and Storage of Blood

From each included individual, five milliliters (mL) of blood during fasting state were obtained by using the Vacutainer system at the time of enrollment in the study. The samples were centrifuged at 2000 rpm for 15 min at 25°C and serum obtained. Samples were then stored at -80°C.

### 2.3. Determination of Serum Catalase Activity (CAT) Concentration

Initially, the total protein content was determined by the bicinchoninic acid (BCA) Protein Assay Kit™ Pierce. Enzyme catalase activity was evaluated by the decrease of the concentration of H_2_O_2_ in the spectrophotometer absorbance measured of 240 nm [24]. A hydrogen peroxide substrate solution 20 mM was prepared with 50 mM phosphate (KH_2_PO_4_), pH 7.4 in Milli-Q water. Then, 10 *μ*L of serum was mixed with 1 mL of substrate solution and measured in a spectrophotometer. A decay curve was built, and the activity was expressed in nmol/min total protein.

### 2.4. Determination of Serum Glutathione (GSH) Concentration

Glutathione concentration was assessed by using the test for determination of nonprotein thiols (NP-SH) [25]. 80 *μ*L of Milli-Q H_2_O and 20 *μ*L of 50% trichloroacetic acid were added into 100 *μ*L of serum for protein precipitation. After that, the sample was centrifuged 30 rpm for 15 min at 4°C. Then, 200 *μ*L aliquots of the supernatant were mixed with 200 *μ*L of 0.4 M TRIS, pH 8.9, and with 5 *μ*L of DTNB (5,5-dithiobis-2-nitro-benzoic acid) in a vortex for 40 s and reading in absorbance of 412 nm. The concentration of GSH was expressed in *μ*mol/mL of blood serum.

### 2.5. Determination of Serum Level of Malondialdehyde (MDA) Concentration

MDA concentration was determined by means of lipidic peroxidation-MDA (Sigma, MAK085) as previously described [25]. Initially, sulfuric acid 42 nM was added to 10 *μ*L of serum in microtube, lightly homogenized and added to 125 *μ*L of phosphotungstic acid. The solution was mixed in a vortex and incubated at room temperature for 5 min. After that, the sample was centrifuged (11.000 rpm) for 5 min at 4°C. The supernatant was discarded and the pellet resuspended with solution of BHT (2 *μ*L of BHT in 100 *μ*L of Milli-Q H_2_O) on ice. 200 *μ*L thiobarbituric acid (TBA) was added to each microtube containing the samples in order to obtain the pattern of the curve (0, 1, 2, 4, 8, 12, 16, and 20 nmol of MDA). Samples were incubated in water bath at 95°C for 60 min and maintained on ice for 10 min. The absorbance (532 nm) was evaluated in 200 *μ*L of each sample. Total serum concentration of MDA was expressed in nmol/mL.

### 2.6. Determination of Serum Superoxide Dismutase (SOD) Activity

The activity of SOD was measured using the photochemic nitro blue tetrazolium (NBT)/riboflavin method as previously described [26]. Briefly, using a 96 well plate, 5 *μ*L of sample, 15 *μ*L of NBT, 30 *μ*L of riboflavin at 10 *μ*M, and 100 *μ*L of buffer (potassium phosphate at 50 mM, EDTA at 0.1 mM, L-methionine 19.5 mM; pH 7.8). The 96 well plate was placed under fluorescent light (20 W) for 15 minutes. The plate was the read using Asys® UVM 340 plate reader at 560 nm wavelength. The results were expressed as units (necessary amount of SOD to decrease NBT by 50% per miligram of protein (U/mg de protein). The total protein amount in each sample was measured using BioRad Protein Assay kit as previously described [27].

### 2.7. Statistical Analysis

The data were analyzed by the SPSS statistical software package version 22.0 (Inc. Chicago, IL). The levels of GSH, CAT, SOD, and MDA were expressed in mean and interquartile range (IQR). Student's two-tailed *t*-test or Mann–Whitney *U* test was adopted based on the results of the Shapiro-Wilk test evaluation. When significant departures from normality were detected, the Mann–Whitney *U* test was adopted. The number of patients per group was calculated using the software G∗Power 3.1.9.2. Normality was checked by using the Shapiro-Wilk test while homogeneity of variance was checked using the Levene test. Kruskal-Wallis test instead of ANOVA was used as our data followed a nonnormal distribution. A post hoc Bonferroni test was performed. A generalized linear model, with Gama distribution and a log link function, was used to assess the correlation between GC and CAT, GSH, MDA, and SOD variables, controlling for sex, age, and smoking and with the effect of interaction between GC group, first-degree relatives of GC, and the control group variables. The level of significance was set at a *p* value ≤ 0.05.

## 3. Results

### 3.1. Patient Characteristics

A total of 155 individuals were included in the study: 50 patients with distal GC (61.00 ± 14.72 mean age), 49 first-degree relatives of GC patients (47.5 ± 11.60 mean age), and 56 subjects in the control group (CG) (48.00 ± 12.39 mean age). The demographic and social features of the patients are outlined in Table 1. The first-degree relatives of GC were similar to controls regarding age, sex, smoking, and alcohol intake. On the other hand, GC patients were significantly different than both controls and first-degree relatives of GC patients with regards to sex, smoking, alcohol use, and age (Table 1). None of the patients in the control group or first-degree relatives of gastric cancer patients had peptic ulcer disease or premalignant histologic findings.

### 3.2. Oxidative Stress Status in Patients with Gastric Cancer

The serum levels of GSH were significantly lower (*p* = 0.001) while catalase activity (*p* = 1.00) and SOD activity (*p* = 0.189) were not significantly different between GC patients and the control group. Lipid peroxidation, measured by concentration of MDA, was significantly higher (*p* = 0.01) in GC patients than in controls as shown in Table 2.

**Figure 1:**
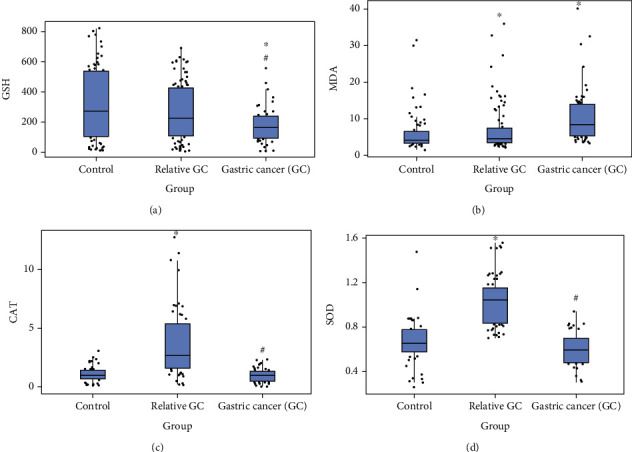
(a) Glutathione serum level (GSH) expressed in *μ*mol/mL; (b) serum catalase activity (CAT) expressed nmol/min; (c) malondialdehyde serum level (MDA), expressed in nmol/mL; and (d) superoxide dismutase, expressed in nmol/mg of protein, of control patients, gastric cancer (GC), and relatives of gastric cancer patients. Detailed univariate and multivariate analysis with *p* values are available in Tables 2 and 4. ∗*p* < 0.05 compared to control; ^#^*p* < 0.05 compared to relative of the GC group. ^§^SOD activity in a subset of patients (control group, *n* = 31; gastric cancer group, *n* = 32; relatives gastric cancer, *n* = 49).

On multivariate analysis adjusting for sex, age, and smoking, the association of lower concentration of GSH as well as higher concentration of MDA in the GC patients than in the other groups (Table 2) remained significant. Median serum values of GSH, MDA, and CAT are represented in Figure 1.

### 3.3. Oxidative Status according to the Cancer Stages

When taking into account gastric cancer stages (I/II or III/IV), the concentration of GSH remained significantly lower and MDA higher in the GC group when compared with the control group. There was no difference in the CAT activity or SOD activity among the groups. No statistical difference was observed when GSH and MDA levels and catalase activity when comparing between the gastric cancer stages I/II and III/IV (Table 3).

### 3.4. Oxidative Status of the First-Degree Relatives of Gastric Cancer Patients

In the multivariate analysis adjusting for sex, age, smoking, and alcohol consumption, the serum concentration of MDA was higher (*p* = 0.003) in first-degree relatives of GC than in controls. However, no difference was observed between GC patients and their first-degree relatives (*p* = 0.66) (Tables 4 and 5). GSH concentration was significantly higher in the first-degree relatives of GC than in the GC patients (*p* = 0.001). Catalase and superoxide dismutase activity were lower in the gastric cancer group (3.82 vs. 0.91; *p* ≤ 0.001 and 1.04 vs. 0.6; *p* ≤ 0.001) compared to their first-degree relatives (Tables 4 and 5), but no difference was observed in comparison with the control group. Median serum values of GSH, MDA, CAT, and SOD are represented in Figure 1.

## 4. Discussion

In this study, we evaluated oxidative stress through concentration of MDA, a lipid peroxidation product, GSH, a nonenzymatic antioxidant, catalase and SOD activity in the serum of GC patients, their first-degree relatives and controls (dyspeptic patients without family history of GC). We demonstrate for the first time that, similarly to GC patients, the first-degree relatives of gastric cancer patients have increased serum oxidative stress without presence of malignant or pre-malignant lesions on upper endoscopy.

The role of oxidative stress in cancer development is complex and not well-defined. Mild to moderate oxidative stress can promote cancer while high levels can suppress survival of cancer cells. A recent study has demonstrated in colorectal cancer patients that oxidative stress may impact the tumour microenvironment and remodeling of tumour stroma by modulating tumour inflammatory infiltration and budding. In addition, they also found a correlation between oxidative stress and tumour staging highlighting the potential for oxidative stress parameters to predict prognosis [28]. Several studies have shown increased serum oxidative stress in patients with malignancy, including breast [29], bladder [30], colorectal [31], and esophageal cancer [32]. However, other studies did not observe such difference in gastric cancer [17]. A study from Turkey showed that gastric mucosa from patients with GC had lower catalase activity and higher concentrations of MDA than in controls [33], while there was no difference in the tissue levels of GSH. In another study from Turkey, increased levels of lipid peroxidation and lower levels of antioxidant enzymes were also observed in the gastric tumor tissues [15]. Others have found similar results when evaluating antioxidant status in the peripheral blood of patients with gastric cancer [34–36]. Although some studies have demonstrated good correlation of increased oxidative stress in the serum and cancerous tissue samples [37], others have shown poor correlation [38]. Therefore, abnormal serum oxidative status in our study may not reflect oxidative stress status in the gastric tissue.

We found that the serum levels of MDA were significantly higher in patients with GC when compared with those of the control group. The association remained significant even after adjustment for age, sex, and smoking. This suggests a high production of ROS and oxidative stress in patients with GC as evidenced by increased lipid peroxidation measured by MDA. In addition, we also found that GSH, a major nonenzymatic antioxidant, was significantly decreased in the GC, in agreement with the studies of others [39]. Interestingly, catalase and superoxide dismutase activities of the first-degree relatives of GC patients were significantly higher than that observed in the GC group, which may represent a compensatory mechanism of oxidant-antioxidant status. In agreement, increased oxidative stress in patients with end-stage heart failure resulted in a compensatory increase of catalase gene expression without change in glutathione peroxidase expression [40].

Genetic variants of oxidant-antioxidant status have been shown to increase the risk of several malignancies including, breast cancer, gliomas, and gastric cancer [41, 42] [43]. It has been also shown that the first-degree relatives of patients with diabetes mellitus have higher levels of oxidative stress than the controls [44] [45] [46]. In this study, we found higher serum levels of lipid peroxidation, as measured by MDA in the first-degree relatives of GC patients compared with controls, even after adjustment for age, sex, and smoking. In agreement, GSH levels in first-degree relatives of GC patients were numerically lower than that of the controls. This finding suggests that oxidative stress status is higher in first-degree relatives than in controls. We hypothesize that this finding may be perhaps explained by a presumably similar genetic background, dietary habits, and infection by more virulent strains of *H. pylori* in GC patients as previously demonstrated by our group [21, 47].

Chronic inflammation associated with *H. pylori* increases oxidative stress, and the more virulent *H. pylori* strains (*cag*A-positive strains) induce oxidative burst in polymorphonuclear cells [48]. Furthermore, more virulent genotype of *H. pylori* strains is associated with higher blood levels of MDA [9, 49]. In this study, *H. pylori* status of the patients was not evaluated; however, previous studies conducted in the same region by our group demonstrated that *H. pylori* infection was highly prevalent in GC (95%), first-degree relative of GC patients (80%), and dyspeptic (75%) patients. We have also previously demonstrated that first-degree relatives of GC patients have high prevalence of pangastritis, precancerous lesions, and they are colonized with the most virulent *H. pylori cag*A and *vac*A-positive genotypes [21, 47].

Limitations of this study include relatively small sample size in each subgroup analyzed, intrinsic differences observed in the age and sex among the different groups studied, and lack of information regarding BMI, dietary intake, and lipid profile. Another limitation of this study is that MDA is known to be susceptible to artifacts as it can react with aldehydes other than MDA [50], and DNA and protein oxidation were not evaluated. Finally, the oxidative stress levels found in serum in our study population may not reflect cellular concentrations, and results should be interpreted with caution.

Strengths of this study include the prospective design, homogenous population from a socioeconomic and geographic standpoint, the exclusion of patients with significant chronic diseases, and robust statistical analysis controlling for confounding variables such as sex, age, smoking, and alcohol use. The novelty of our findings is another strength of this study since it is hypothesis generating and can allow for future studies looking at mechanistic causality of ROS in first-degree relative of GC patient. This can potentially identify future biomarkers for screening and early detection of GC and potential targets for individualized treatment. Future directions include validation of our findings in a larger longitudinal cohort of patients to determine if oxidative stress status may predict development of gastric cancer in relatives of gastric cancer patients or in the general population and performing a more robust assessment of oxidative stress to include markers of DNA and protein oxidation. In addition, our findings also support further studies to determine if there is genetic predisposition to abnormal oxidative stress response that may increase susceptibility to the development of gastric cancer.

## 5. Conclusion

In conclusion, this study shows that oxidative stress was more markedly observed in the GC patient, regardless of the tumor stages, than in first-degree relatives of GC patients and in controls. Notably, we demonstrated for the first time, to the best of our knowledge, increased lipid peroxidation in first-degree relatives of GC patients, similarly to that observed in patients with gastric cancer. Because MDA is a product of polyunsaturated fatty acids and has been considered to be mutagenic and carcinogenic, it may contribute to the increased risk of GC in this group of individuals. Demonstration of increased oxidative stress may be relevant for identification of groups at increased risk of gastric cancer. Further studies are warranted to confirm the results observed in this study and to add more data on the role of oxidative stress in increasing the gastric cancer development in the first-degree relatives of GC patients.

## Figures and Tables

**Table 1:** Clinical characteristics of the sample population.

Characteristics	Gastric cancer (*N* = 50)	Dyspeptic controls (*N* = 56)	Relatives of gastric cancer (*N* = 49)	*p* value
Age (yrs), mean	61	48	47.5	0.00^a^ 0.863^b^
Gender, *n* (%)				
Male	34 (68)	13 (23.2)	15 (30.6)	0.392^a^
Female	16 (32)	43 (76.8)	34 (69.4)	0.00^b^
Alcohol use, *n* (%)	28 (56)	17 (30.3)	11 (22.4)	0.08^a^ 0.361^b^
Chronic smoking, *n* (%)	29 (58)	8 (14.2)	0	0.00^a^ 0.06^b^
Education level				
< 9 years	42 (84)	15 (26.8)	20 (40.8)	0.000^a^
≥ 9 years	8 (16)	41 (73.2)	29 (59.2)	0.153^b^
Income				
< US$400	44 (88)	40 (71)	37 (75.5)	0.036^a^
≥ US$ 400	6 (12)	16 (28.5)	12 (24.4)	0.597^b^
Lauren type				
Intestinal	38 (76)	—	—	—
Diffuse	12 (24)	—	—	—
TNM stage				
I	12 (24)	—	—	—
II	8 (16)	—	—	—
III	6 (12)	—	—	—
IV	24 (48)	—	—	—

^a^Controls vs. gastric cancer. ^b^Controls vs. relatives of gastric cancer.

**Table 2:** Comparison of serum GSH concentration, CAT, MDA concentration, between controls and gastric cancer patients.

Univariate	CG	GC	*p*
GSH	337.76 (112.18-561)	125.72 (46.29-220.41)	<0.001
CAT	0.97 (0.69-1.42)	0.95 (0.43-1.33)	0.355
MDA	3.85 (3.33-6.47)	6.32 (3.94-12.13)	0.011
SOD	0.66 (0.58-0.77)	0.6 (0.48-0.73)	0.163
Multivariate∗	CG	GC	*p*
GSH	325.5 (309.25-380.42)	137.84 (125.98-157.91)	<0.001
CAT	1.04 (0.92-1.15)	0.9 (0.79-1.05)	0.329
MDA	5.44 (5.16-5.57)	8.93 (8.53-9.43)	0.001
SOD	0.67 (0.67-0.68)	0.6 (0.59-0.61)	0.189

∗Values estimated for generalized linear model gama-log: intercept, sex, smoking, cancer, and age. Data presented as median (25th percentile-75th percentile). CG control group and GC gastric cancer group.

**Table 3:** Comparison of serum GSH concentration, CAT activity, MDA, and SOD activity between control group and gastric cancer stage I, II and III, IV.

	Control	I e II	III e IV	*p*
GSH	337.76 (112.18-561)	78.41 (24.77-233.35)	140.41 (77.47-206.88)	<0.001^a,b^
CAT	0.97 (0.69-1.42)	1.11 (0.55-1.49)	0.85 (0.37-1.3)	0.381
MDA	3.85 (3.33-6.47)	5.82 (3.14-12.51)	6.58 (4.45-11.83)	0.028^b^
SOD	0.64 (0.54-0.81)	0.55 (0.44–0.67)	0.49 (0.47–0.66)	0.254

^a^ *p* < 0.05 GC stage I, II vs. control. ^b^*p* < 0.005 GC stage III, IV vs. control.

**Table 4:** Comparison of serum GSH concentration, CAT activity, MDA concentration, and SOD activity between controls and familial gastric cancer patients.

Univariate	CG	Relatives of GC	*p*
GSH	337.76 (112.18-561)	225.12 (117.47-438.06)	0.147
CAT	0.97 (0.69-1.42)	2.71 (1.53-5.38)	<0.001
MDA	3.85 (3.33-6.47)	5.37 (3.6-10.82)	0.051
SOD	0.66 (0.58-0.77)	1.04 (0.83-1.15)	<0.001
Multivariate∗	CG	Relatives of GC	*p*
GSH	313.36 (312.45-373.49)	256.29 (255.88-305.12)	0.258
CAT	1.05 (0.97-1.1)	3.81 (3.51-3.93)	<0.001
MDA	5.15 (4.93-5.79)	7.95 (7.78-8.92)	0.003
SOD	0.67 (0.67-0.68)	1.04 (1-1.05)	<0.001

∗Values estimated for generalized linear model gama-log: intercept, sex, smoking, cancer, and age. Data presented as median (25th percentile-75th percentile). CG control group and relatives of gastric cancer.

**Table 5:** Comparison of serum GSH concentration, CAT activity, MDA concentration, and SOD activity and between gastric cancer patients and familial gastric cancer patients.

Univariate	Relatives of GC	GC	*p*
GSH	225.12 (117.47-438.06)	125.72 (46.29-220.41)	0.001
CAT	2.71 (1.53-5.38)	0.95 (0.43-1.33)	<0.001
MDA	5.37 (3.6-10.82)	6.32 (3.94-12.13)	0.627
SOD	1.04 (0.83-1.15)	0.6 (0.48-0.73)	<0.001
Multivariate∗	Relatives of GC	GC	*p*
GSH	262.51 (255.84-275.57)	144.64 (127.23-152.63)	0.018
CAT	3.82 (3.47-4.05)	0.91 (0.78-1.09)	<0.001
MDA	7.58 (7.25-9.12)	9.25 (7.99-10.46)	0.659
SOD	1.04 (1-1.05)	0.6 (0.59-0.61)	<0.001

∗Values estimated for generalized linear model gama-log: intercept, sex, smoking, cancer, and age. Data presented as median (25th percentile-75th percentile). GC: gastric cancer.

## Data Availability

The datasets used and/or analyzed during the current study are available from the corresponding author on reasonable request.

## References

[1] Bray F., Ferlay J., Soerjomataram I., Siegel R. L., Torre L. A., Jemal A. (2018). Global cancer statistics 2018: GLOBOCAN estimates of incidence and mortality worldwide for 36 cancers in 185 countries. *CA: a Cancer Journal for Clinicians*.

[2] Asplund J., Kauppila J. H., Mattsson F., Lagergren J. (2018). Survival trends in gastric adenocarcinoma: a population-based study in Sweden. *Annals of Surgical Oncology*.

[3] Braga L., Ramos A. N., Braga Neto M. B. (2019). Unequal burden of mortality from gastric cancer in Brazil and its regions, 2000-2015. *Gastric Cancer*.

[4] Etemadi A., Safiri S., Sepanlou S. G. (2020). The global, regional, and national burden of stomach cancer in 195 countries, 1990-2017: a systematic analysis for the Global Burden of Disease study 2017. *Gastroenterología y Hepatología*.

[5] Klaunig J. E. (2019). Oxidative stress and cancer. *Current Pharmaceutical Design*.

[6] Kamendulis L. M., Wu Q., Sandusky G. E., Hocevar B. A. (2014). Perfluorooctanoic acid exposure triggers oxidative stress in the mouse pancreas. *Toxicology Reports*.

[7] Prasad S., Gupta S. C., Pandey M. K., Tyagi A. K., Deb L. (2016). Oxidative stress and cancer: advances and challenges. *Oxidative Medicine and Cellular Longevity*.

[8] Bhattacharyya A., Chattopadhyay R., Mitra S., Crowe S. E. (2014). Oxidative stress: an essential factor in the pathogenesis of gastrointestinal mucosal diseases. *Physiological Reviews*.

[9] Butcher L. D., den Hartog G., Ernst P. B., Crowe S. E. (2017). Oxidative Stress Resulting From _Helicobacter pylori_ Infection Contributes to Gastric Carcinogenesis. *Cellular and Molecular Gastroenterology and Hepatology*.

[10] Pasupathi P., Saravanan G., Chinnaswamy P., Bakthavathsalam G. (2009). Effect of chronic smoking on lipid peroxidation and antioxidant status in gastric carcinoma patients. *Indian Journal of Gastroenterology*.

[11] He L., He T., Farrar S., Ji L., Liu T., Ma X. (2017). Antioxidants maintain cellular redox homeostasis by elimination of reactive oxygen species. *Cellular Physiology and Biochemistry*.

[12] Ayala A., Munoz M. F., Arguelles S. (2014). Lipid peroxidation: production, metabolism, and signaling mechanisms of malondialdehyde and 4-hydroxy-2-nonenal. *Oxidative Medicine and Cellular Longevity*.

[13] Qing X., Shi D., Lv X., Wang B., Chen S., Shao Z. (2019). Prognostic significance of 8-hydroxy-2′-deoxyguanosine in solid tumors: a meta-analysis. *BMC Cancer*.

[14] Kruk J., Aboul-Enein H. Y. (2017). Reactive oxygen and nitrogen species in carcinogenesis: implications of oxidative stress on the progression and development of several cancer types. *Mini Reviews in Medicinal Chemistry*.

[15] Kekec Y., Paydas S., Tuli A., Zorludemir S., Sakman G., Seydaoglu G. (2009). Antioxidant enzyme levels in cases with gastrointesinal cancer. *European Journal of Internal Medicine*.

[16] Borrego S., Vazquez A., Dasí F. (2013). Oxidative stress and DNA damage in human gastric carcinoma: 8-oxo-7'8-dihydro-2'-deoxyguanosine (8-oxo-dG) as a possible tumor marker. *International Journal of Molecular Sciences*.

[17] Ma Y., Zhang L., Rong S. (2013). Relation between gastric cancer and protein oxidation, DNA damage, and lipid peroxidation. *Oxidative Medicine and Cellular Longevity*.

[18] Sitarz R., Skierucha M., Mielko J., Offerhaus G. J. A., Maciejewski R., Polkowski W. P. (2018). Gastric cancer: epidemiology, prevention, classification, and treatment. *Cancer Management and Research*.

[19] Brenner H., Arndt V., Sturmer T., Stegmaier C., Ziegler H., Dhom G. (2000). Individual and joint contribution of family history andHelicobacter pylori infection to the risk of gastric carcinoma. *Cancer*.

[20] Choi I. J., Kim C. G., Lee J. Y. (2020). Family history of gastric cancer and *Helicobacter pylori* Treatment. *The New England Journal of Medicine*.

[21] Queiroz D. M., Silva C. I., Goncalves M. H. (2012). Higher frequency of cagA EPIYA-C phosphorylation sites in H. pylori strains from first-degree relatives of gastric cancer patients. *BMC Gastroenterology*.

[22] Lauren P. (1965). The two histological main types of gastric carcinoma: diffuse and so-called intestinal-type CARCINOMA. *Acta Pathologica et Microbiologica Scandinavica*.

[23] Edge S. B., Compton C. C. (2010). The American joint committee on cancer: the 7th edition of the AJCC cancer staging manual and the future of TNM. *Annals of Surgical Oncology*.

[24] Beers R. F., Sizer I. W. (1952). A spectrophotometric method for measuring the breakdown of hydrogen peroxide by catalase. *The Journal of Biological Chemistry*.

[25] Sedlak J., Lindsay R. H. (1968). Estimation of total, protein-bound, and nonprotein sulfhydryl groups in tissue with Ellman's reagent. *Analytical Biochemistry*.

[26] Beauchamp C., Fridovich I. (1971). Superoxide dismutase: improved assays and an assay applicable to acrylamide gels. *Analytical Biochemistry*.

[27] Lowry O. H., Rosebrough N. J., Farr A. L., Randall R. J. (1951). Protein measurement with the Folin phenol reagent. *The Journal of Biological Chemistry*.

[28] Zińczuk J., Maciejczyk M., Zaręba K. (2020). Pro-oxidant enzymes, redox balance and oxidative damage to proteins, lipids and DNA in colorectal cancer tissue. Is oxidative stress dependent on tumour budding and inflammatory infiltration?. *Cancers*.

[29] Feng J. F., Lu L., Dai C. M. (2016). Analysis of the diagnostic efficiency of serum oxidative stress parameters in patients with breast cancer at various clinical stages. *Clinical Biochemistry*.

[30] Gecit I., Aslan M., Gunes M. (2012). Serum prolidase activity, oxidative stress, and nitric oxide levels in patients with bladder cancer. *Journal of Cancer Research and Clinical Oncology*.

[31] Wu R., Feng J., Yang Y. (2017). Significance of serum total oxidant/antioxidant status in patients with colorectal cancer. *PLoS One*.

[32] Huang Q., Feng J., Wu R. (2017). Total oxidant/antioxidant status in sera of patients with esophageal cancer. *Medical Science Monitor*.

[33] Batcioglu K., Mehmet N., Ozturk I. C. (2006). Lipid peroxidation and antioxidant status in stomach cancer. *Cancer Investigation*.

[34] Pasupathi P., Saravanan G., Chinnaswamy P., Bakthavathsalam G. (2009). Glutathione, glutathione-dependent enzymes and antioxidant status in gastric carcinoma patients. *Journal of Applied Biomedicine*.

[35] Bakan E., Taysi S., Polat M. F. (2002). Nitric oxide levels and lipid peroxidation in plasma of patients with gastric cancer. *Japanese Journal of Clinical Oncology*.

[36] Ilhan N., Ilhan N., Ilhan Y., Akbulut H., Kucuksu M. (2004). C-reactive protein, procalcitonin, interleukin-6, vascular endothelial growth factor and oxidative metabolites in diagnosis of infection and staging in patients with gastric cancer. *World Journal of Gastroenterology*.

[37] Mehdi M., Menon M. K. C., Seyoum N., Bekele M., Tigeneh W., Seifu D. (2018). Blood and tissue enzymatic activities of GDH and LDH, index of glutathione, and oxidative stress among breast cancer patients attending referral hospitals of Addis Ababa, Ethiopia: hospital-based comparative cross-sectional study. *Oxidative Medicine and Cellular Longevity*.

[38] Yeh C. C., Hou M. F., Wu S. H. (2006). A study of glutathione status in the blood and tissues of patients with breast cancer. *Cell Biochemistry and Function*.

[39] Scibior D., Skrzycki M., Podsiad M., Czeczot H. (2008). Glutathione level and glutathione-dependent enzyme activities in blood serum of patients with gastrointestinal tract tumors. *Clinical Biochemistry*.

[40] Dieterich S., Bieligk U., Beulich K., Hasenfuss G., Prestle J. (2000). Gene expression of antioxidative enzymes in the human heart. *Circulation*.

[41] Rodrigues P., de Marco G., Furriol J. (2014). Oxidative stress in susceptibility to breast cancer: study in Spanish population. *BMC Cancer*.

[42] Geng R., Chen Z., Zhao X. (2014). Oxidative stress-related genetic polymorphisms are associated with the prognosis of metastatic gastric cancer patients treated with epirubicin, oxaliplatin and 5-fluorouracil combination chemotherapy. *PLoS One*.

[43] Zhao P., Zhao L., Zou P. (2012). Genetic oxidative stress variants and glioma risk in a Chinese population: a hospital-based case-control study. *BMC Cancer*.

[44] Gómez García A., Rodríguez M. R., Gómez Alonso C., Ochoa D. Y. R., Alvarez Aguilar C. (2015). Myeloperoxidase is associated with insulin resistance and inflammation in overweight subjects with first-degree relatives with type 2 diabetes mellitus. *Diabetes and Metabolism Journal*.

[45] Baig S., Shabeer M., Parvaresh Rizi E. (2020). Heredity of type 2 diabetes confers increased susceptibility to oxidative stress and inflammation. *BMJ Open Diabetes Research & Care*.

[46] Sathiyapriya V., Selvaraj N., Nandeesha H., Bobby Z., Agrawal A., Pavithran P. (2007). Enhanced glycation of hemoglobin and plasma proteins is associated with increased lipid peroxide levels in non-diabetic hypertensive subjects. *Archives of Medical Research*.

[47] Motta C. R., Cunha M. P., Queiroz D. M. (2008). Gastric precancerous lesions and *Helicobacter pylori* infection in relatives of gastric cancer patients from northeastern Brazil. *Digestion*.

[48] Ding S. Z., Minohara Y., Fan X. J. (2007). Helicobacter pyloriInfection induces oxidative stress and programmed cell death in human gastric epithelial cells. *Infection and Immunity*.

[49] Tiwari S. K., Manoj G., Sharma V. (2010). Relevance of helicobacter pylori genotypes in gastric pathology and its association with plasma malondialdehyde and nitric oxide levels. *Inflammopharmacology*.

[50] Katerji M., Filippova M., Duerksen-Hughes P. (2019). Approaches and methods to measure oxidative stress in clinical samples: research applications in the cancer field. *Oxidative Medicine and Cellular Longevity*.

